# Recurrent Squamous Cell Carcinoma of the Eyelid Presenting as Trigeminal Neuralgia

**DOI:** 10.7759/cureus.932

**Published:** 2016-12-16

**Authors:** Nicole Spitzer, Naazli Shaikh, Leah Strickland, Son Ho

**Affiliations:** 1 Ophthalmology, University of Central Florida College of Medicine; 2 Department of Pathology, VA Hospital Tampa; 3 Ophthalmology, Orlando VA Medical Center

**Keywords:** squamous cell carcinoma, orbital apex syndrome, trigeminal neuralgia

## Abstract

This paper describes two patients with squamous cell carcinoma (SCC) of the periocular and periorbital skin who presented with trigeminal neuralgia. Both patients had previous cutaneous SCC of the scalp treated successfully with surgical resection but later presented with neuro-ophthalmic findings suggesting perineural invasion (PNI) of SCC. PNI of SCC in the periocular skin or orbit can lead to devastating effects if malignant cells seed into the orbit and adjacent cranial nerves as our two patients developed an orbital apex syndrome. Patients with a history of SCC of the scalp and forehead who later develop neuro-ophthalmological deficits or patients with persistent ocular symptoms should, in particular, be followed with a low threshold for cutaneous SCC or PNI of recurrent disease. SCC metastasizing into the periocular tissues and orbit by neural invasion is rare and carries a poor prognosis. The urgency for a prompt diagnosis and evaluation by a multidisciplinary team is warranted to prevent untoward outcomes of this skin cancer.

## Introduction

Squamous cell carcinoma (SCC) is the second most common eyelid malignancy in Caucasians and accounts for less than 5% of malignant eyelid neoplasms [1]. Most cases of cutaneous SCC are locally resectable and highly curative, although local and distant metastases develop in less than 1-5% of cases [1-2]. Perineural invasion (PNI) of SCC is highly associated with an increased rate of local recurrence (16-47.2%), metastasis (10-50%), and even death [2]. The most common symptoms associated with nerve invasion of SCC include pain, weakness, or numbness with deficits of the trigeminal (V) and facial (VII) nerves being the most commonly reported with cancers of the head and neck [3]. Lesions of the forehead and brow tend to have a higher association with PNI in about 3-14% of squamous cell carcinomas; yet, many clinicians remain unaware of such neurotropic spread of tumors [3-4].

Patients may be asymptomatic initially, with cranial nerve deficits being the first presenting symptoms as the tumor invades the orbit and adnexa. Such patients may present to the ophthalmologist first, which necessitates awareness and diligent follow-up of this condition to avoid delayed diagnosis. Two cases of perineural spread of squamous cell carcinoma of the eyelid and periocular skin are described, all initially diagnosed with trigeminal neuralgia and later developing orbital apex syndromes indicating metastatic spread. 

## Case presentation

Informed patient consent was obtained from both patients for their treatment.

### Case A

**Figure 1:**
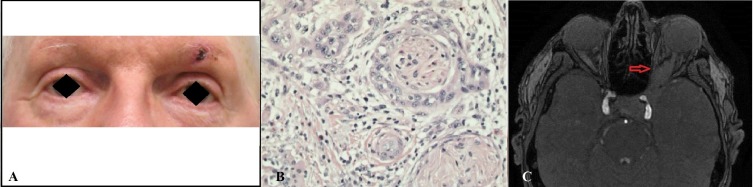
(A) A 79-year-old man presenting with left-sided eyelid pain and an ulcerative brow lesion. (B) Excisional biopsy showing the histological appearance of SCC with perineural invasion (H&E 100x) of the brow lesion. (C) Axial MRI revealing an abnormal enhancing mass (red arrow) of the left cavernous sinus surrounding the left cavernous carotid artery extending into the left orbit anteriorly encasing the left optic nerve. SCC: squamous cell carcinoma

A 79-year-old male with prior excision of a cutaneous SCC of the left temple was referred to the eye clinic with mild left eyelid ptosis, trigeminal neuralgia along the V1 dermatome, and an ulcerative lesion on the left brow (Figure 1A). There was a suspicion for recurrence of SCC, and he underwent a wide excision with a frozen section of the brow lesion. Biopsy showed invasive, well-differentiated SCC with an aggressive growth pattern, along with PNI and clear margins (Figure 1C). The patient was stable for one year until he developed left facial numbness, complete left eye ptosis, loss of vision, and ophthalmoplegia. An MRI of the brain and orbits revealed an abnormal expansion and enhancing mass of the left cavernous sinus encasing the left cavernous carotid artery, extending into the left orbit anteriorly, encasing the left optic nerve, and infiltrating the inferior, medial, and superior recti muscles (Figure 1B). These abnormalities of cranial nerves 2, 3, 4, 5 (V1 and V2), and 6 indicated an orbital apex syndrome. The patient subsequently received stereotactic radiation treatment and chemotherapy; however, he died six months later.

### Case B

**Figure 2:**
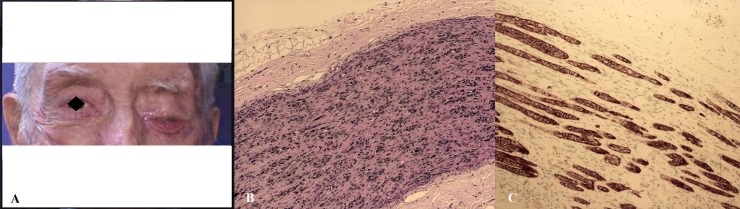
(A) An 80-year-old man presenting with left-sided facial droop, complete eyelid ptosis, and marked impairment of left ocular motility. (B) A photomicrographic biopsy of the supraorbital branch of the facial nerve revealing PNI of poorly differentiated squamous cell carcinoma (H&E 20x). (C) Positive staining for CK5/6 (20x). PNI: perineural invasion; CK 5/6: cytokeratin 5/6

This 81-year-old male with SSC of the forehead, who previously underwent Moh’s surgery for excision, presented with left-sided facial pain, numbness, and pruritus, which were initially diagnosed as trigeminal neuralgia. His symptoms further progressed to left-sided facial droop, complete eyelid ptosis, marked impairment of left ocular motility, and left-sided hearing loss that had gradually progressed over two years (Figure 2A). An orbital apex syndrome was diagnosed, and an MRI of the brain and orbits revealed pathological enhancement within the superior orbital fissure, foramen rotundum, and foramen ovale with extension into the superior orbital fissure. A biopsy of the supraorbital branch of the facial nerve revealed PNI of poorly differentiated squamous cell carcinoma positive for cytokeratin (CK) 5/6 (Figure 2B-C). A tumor board regarded his Stage IV SCC to be unresectable and that he was a poor candidate for radiation therapy due to his complicated medical history and diffuse spread of the tumor. The patient was started on systemic chemotherapy for palliative care and subsequently died within one year.

## Discussion

Perineural spread of SCC is a rare but devastating manifestation of head and neck cancers. Certain histopathological features like location, size, depth, histological features, immunosuppression, and perineural invasion constitute “high-risk” cutaneous SCC, which is associated with more aggressive tumor behavior and portends a higher rate of metastasis and recurrent disease. Orbital invasion, in particular, has a poor prognosis and, in most cases, results from a primary tumor in the forehead/scalp region. Such metastatic spread is one of the hallmarks of the aggressiveness of squamous cell carcinoma. Fewer than 5% of squamous cell carcinomas are confirmed pathologically as perineural spread, and of these, only 30-40% of patients have phenotypic abnormalities on presentation [4-5]. The cases presented in this report, therefore, validate the necessity of physician vigilance for the metastatic potential of skin cancers and underscore the need for early diagnosis, as the five-year survival rate once clinical features are apparent is a dismal 20-30% [1].

The confirmation of PNI despite suggestive signs and symptoms can prove difficult, and it may not be until disease develops within the orbit, cavernous sinus, and/or nerves/foramina that a diagnosis can be supported. The most common clinical findings are paresthesia due to the involvement of the V1 branch of the trigeminal nerve and ophthalmoplegia from the involvement of the oculomotor nerve [1, 3-4]. Patients presenting with ophthalmic findings with the involvement of multiple cranial nerves should alert the clinician to the development of an orbital apex syndrome, which is characterized by ophthalmoplegia, proptosis, ptosis, hypesthesia of the forehead, and vision loss. Tumor invasion to cause this syndrome is usually centripetal, through branches of cranial nerve V1, spreading towards the central nervous system, but may become centrifugal once it reaches a junctional point, such as the trigeminal ganglion [6]. The microscopic appearances suggest that the tumor extends along the perineural space and penetrates through the epineurium to gain access to the orbital connective tissue [7].

Diagnosis of perineural spread may be helped with imaging, such as MRI with contrast, as our two patients demonstrated obvious lesions; however, this was in the setting of an orbital apex syndrome. The presentation of SCC has been described on imaging (CT and MRI) and linked to PNI; the cases with radiographic evidence of perineural spread have been shown to be associated with a poorer prognosis (50% five-year survival rate) than imaging-negative disease (86% five-year survival rate) [3, 8]. Treatment recommendations for such metastatic spread of SCC remain controversial. Aggressive approaches involving the early use of radiotherapy and radical surgery have been postulated to be of some benefit; however, early intervention may ultimately be the crux of treatment. Both patients were being seen by physicians across various disciplines, which may have delayed diagnosis, and is an inherent challenge in elderly cancer patients. It is, therefore, crucial that a low threshold for diagnosis be maintained for patients who have a history of cutaneous SCC of the scalp and forehead with subsequent development of neurological symptoms around the face and eye. 

## Conclusions

The presentation of trigeminal neuralgia and/or ophthalmic sensory and/or motor deficits in patients with a history of SCC of the face and scalp is suggestive of perineural invasion and metastatic disease. These characteristics highlight advanced disease and should prompt an urgent biopsy and imaging studies to determine the extent of the disease and a multi-disciplinary approach to treatment.
